# Microporous polysaccharide multilayer coated BCP composite scaffolds with immobilised calcitriol promote osteoporotic bone regeneration both *in vitro* and *in vivo*: Erratum

**DOI:** 10.7150/thno.61641

**Published:** 2021-04-20

**Authors:** Qian Tang, Zhichao Hu, Haiming Jin, Gang Zheng, XingFang Yu, Gang Wu, Haixiao Liu, Zhenzhong Zhu, Huazi Xu, Changqing Zhang, Liyan Shen

**Affiliations:** 1 Key Laboratory of Orthopaedics of Zhejiang Province, Department of Orthopaedics, The Second Affiliated Hospital and Yuying Children's Hospital of Wenzhou Medical University, 109, Xueyuanxi road, 325027 Wenzhou, China; 2 The second School of Medicine, Wenzhou Medical University, 109, Xueyuanxi road, 325027 Wenzhou, China; 3 Department of Orthopaedic Surgery Shanghai Jiao Tong University Affiliated Sixth People's Hospital, 600 Yishan Road, Shanghai, 200233, China.; 4 Department of Oral Implantology and Prosthetic Dentistry, Academic Centre for Dentistry Amsterdam (ACTA), Vrije University Amsterdam and University of Amsterdam, Amsterdam, Nord-Holland, the Netherlands

The authors regret that the image of BCP group without Cal in 12 W in the Fig. 7B was wrongly attached due to their carelessness in integrating figures. The correct version is shown below. This image substitution would not affect any results presented in the originally-published version, nor the corresponding text description and the conclusion of the paper. The authors apologize for any inconvenience or misunderstanding that this error may have caused.

## Figures and Tables

**Figure 7:**
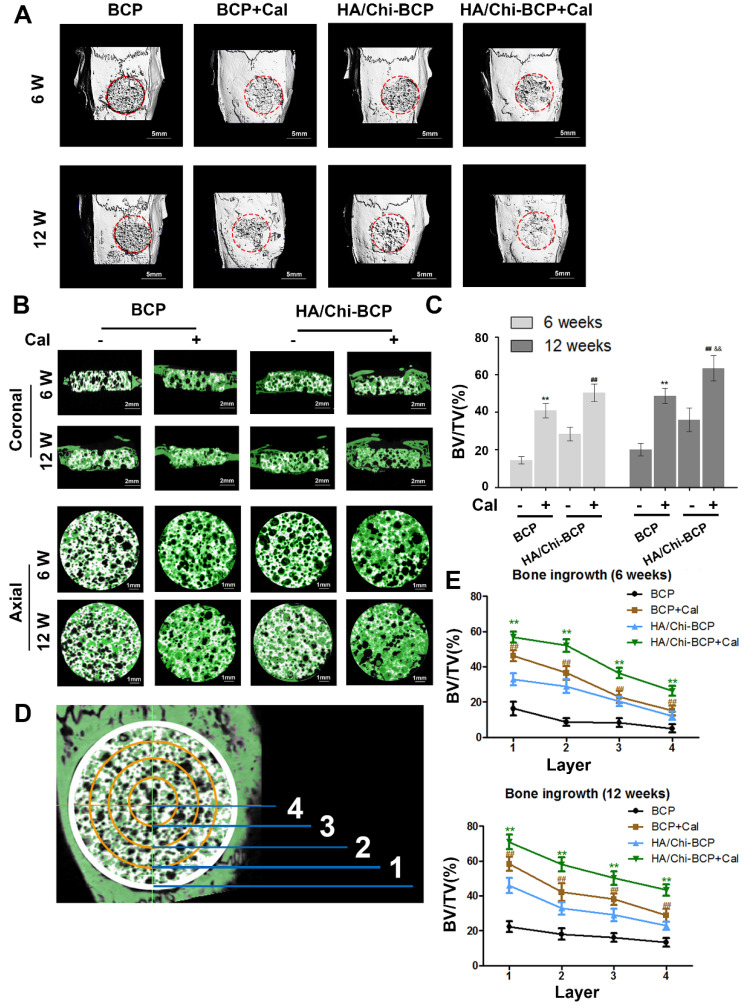
Micro-CT analysis of the effects of various BCP scaffolds on new bone formation in the critical-size bone defect model of OVX rats. (A) Three-dimensional reconstruction of micro-CT images of the various scaffolds implanted in the rat calvarium at 6 and 12 weeks (scale bars: 5 mm). (B) Two-dimensional reconstruction of micro-CT images of various scaffolds implanted in the rat calvarium at 6 and 12 weeks (the white color component shows the remaining scaffold, bone that grew around and into the scaffolds is labelled in green) (scale bars: 2 mm for coronal images and 1 mm for axial images). (C) Regenerated bone volumes on the various scaffolds were quantified as bone volume divided by total volume (BV/TV). (D) General sketch of the scaffold, which was divided into four layers. (E) Percentages of bone volume regenerated into the scaffold in different layers from the edge to the center. Data are presented as the mean ± S.D. Significant differences among scaffold groups are indicated as ** P < 0.01, * P < 0.05, compared with BCP; ## P < 0.01, # P < 0.05 compared with HA/Chi-BCP, and && P < 0.01, & P < 0.05 compared with BCP+Cal; for each group, n = 5.
